# Specific antibody binding to the APP_672–699_ region shifts APP processing from *α*- to *β*-cleavage

**DOI:** 10.1038/cddis.2014.336

**Published:** 2014-08-14

**Authors:** S Li, J Deng, H Hou, J Tian, B Giunta, Y Wang, D Sawmiller, A Smith, P R Sanberg, D Obregon, T Mori, J Tan

**Affiliations:** 1 Rashid Laboratory for Developmental Neurobiology, Silver Child Development Center, Department of Psychiatry and Behavioral Neurosciences, Morsani College of Medicine, University of South Florida, Tampa, FL, USA; 2 Center for Translational Research of Neurology Diseases, First Affiliated Hospital, Dalian Medical University, Dalian, China; 3 Department of Neurology, Daping Hospital, The Third Military Medical University, Chongqing, China; 4 James A. Haley Veterans' Hospital, Tampa, FL, USA; 5 Neuroimmunology Laboratory, Department of Psychiatry and Behavioral Neurosciences, Morsani College of Medicine, University of South Florida, Tampa, FL, USA; 6 Center of Excellence for Aging and Brain Repair, Department of Neurosurgery and Brain Repair, Morsani College of Medicine, University of South Florida, Tampa, FL, USA; 7 Departments of Biomedical Sciences and Pathology, Saitama Medical Center and Saitama Medical University, Kawagoe, Japan

## Abstract

Alzheimer's disease (AD), a progressive neurodegenerative disorder that is the most common cause of dementia in the elderly, is characterized by the accumulation of amyloid-*β* (A*β*) plaques and neurofibrillary tangles, as well as a progressive loss of synapses and neurons in the brain. The major pertinacious component of amyloid plaques is A*β*, a variably sized peptide derived from the integral membrane protein amyloid precursor protein (APP). The A*β* region of APP locates partly within its ecto- and trans-membrane domains. APP is cleaved by three proteases, designated as *α*-, *β*-, and *γ*-secretases. Processing by *β*- and *γ*-secretase cleaves the N- and C-terminal ends of the A*β* region, respectively, releasing A*β*, whereas *α*-secretase cleaves within the A*β* sequence, releasing soluble APP*α* (sAPP*α*). The *γ*-secretase cleaves at several adjacent sites to yield A*β* species containing 39–43 amino acid residues. Both *α*- and *β*-cleavage sites of human wild-type APP are located in APP_672–699_ region (ectodomain of *β*-C-terminal fragment, ED-*β*-CTF or ED-C99). Therefore, the amino acid residues within or near this region are definitely pivotal for human wild-type APP function and processing. Here, we report that one ED-C99-specific monoclonal antibody (mAb_ED-C99_) blocks human wild-type APP endocytosis and shifts its processing from *α*- to *β*-cleavage, as evidenced by elevated accumulation of cell surface full-length APP and *β*-CTF together with reduced sAPP*α* and *α*-CTF levels. Moreover, mAb_ED-C99_ enhances the interactions of APP with cholesterol. Consistently, intracerebroventricular injection of mAb_ED-C99_ to human wild-type APP transgenic mice markedly increases membrane-associated *β*-CTF. All these findings suggest that APP_672–699_ region is critical for human wild-type APP processing and may provide new clues for the pathogenesis of sporadic AD.

Abnormal functioning and/or processing of amyloid precursor protein (APP), a type I membrane protein, has a pivotal role in the pathogenesis of Alzheimer's disease (AD).^1, 2, 3^ APP is cleaved by three proteases, designated as *α*-, *β*-, and *γ*-secretases (Supplementary Figure S1). The major fraction (>90%) of wild-type APP is proteolyzed by *α*-secretase that cleaves wild-type APP between residues APP_687_ and APP_688_ within the amyloid-*β* (A*β*) sequence, releasing soluble APP*α* (sAPP*α*) and *α*-C-terminal fragment (*α*-CTF, C83). Only a minority (<10%) of all wild-type APP molecules undergo *β*-cleavage at the *β*-cleavage site (between residues APP_671_ and APP_672_) generating sAPP*β* and *β*-CTF (C99), the latter of which is subsequently processed by *γ*-secretase complex to generate a mixture of A*β* peptides primarily 40 or 42 residues in length (A*β*_1-40/42_).^4, 5^ The *β*-secretase cleaves APP in addition at a *β*′-site (between residues APP_681_ and APP_682_) to generate C89 that is further processed by *γ*-secretase to produce truncated A*β*_11–40/42_ species.^6^

Both *α*- and *β*-cleavage sites of wild-type APP are located in APP_672–699_ region (the ectodomain of *β*-CTF, ED-*β*-CTF, or ED-C99; Supplementary Figure S1). Therefore, the amino acid residues within or near this region are definitely pivotal for wild-type APP function and processing. Previous studies have identified that mutation in ED-C99 region can affect the physiological processing of APP and contribute to pathological features of familial AD (fAD). For example, Swedish APP carrying APP_670/671_ mutation (KM→NL) is cleaved by *β*-secretase over 50-fold more efficiently than wild-type APP.^7^ APP_673_ mutation (A→V) and APP_693_ mutation (E→G) can enhance A*β* production and accelerate formation of amyloid fibrils.^8, 9, 10^ APP_682_ mutation (E→K) blocks APP *β*′-site and shifts cleavage to *β*-site, thus increasing A*β*_1–40/42_ production.^6^ Although sporadic AD (sAD), the more common type of AD comprising 90 to 95% of all AD cases, lacks mutations in the *APP* gene, region-specific protein modifications within the ED-C99 region may affect wild-type APP processing similarly to APP gene mutations. For example, phosphorylation of ED-C99 at the threonine 687 (of APP_770_ isoform, or corresponding threonine 668 of APP_751_ isoform; Supplementary Figure S1) facilitates APP processing by *γ*-secretase.^11^ Therefore, the elucidation of potential influences of region-specific modifications, induced by either endogenous or exogenous molecules, on wild-type APP processing would be especially critical for clarifying the mechanisms underlying the pathogenesis of sAD.

To confirm this hypothesis, we used one mouse monoclonal antibody specifically recognizing ED-C99 (mAb_ED-C99_) with its epitope at APP_674–679_ (Supplementary Figure S1). The influences of mAb_ED-C99_ binding on human wild-type APP processing were evaluated *in vitro* using Chinese hamster ovary cells expressing human wild-type APP (CHO/APP_wt_ cells) and cortical neurons derived from human wild-type APP transgenic (TgAPP_wt_) mice. The *in vitro* effects of ED-C99 binding with mAb_ED-C99_ on wild-type APP processing were further evaluated and confirmed *in vivo* using TgAPP_wt_ mice and 5 × FAD transgenic mice (Tg6799 line).

## Results

### Specific binding of mAb_ED-C99_ inhibits *α*- but promotes *β*-cleavage of human wild-type APP

**Figure 1:**
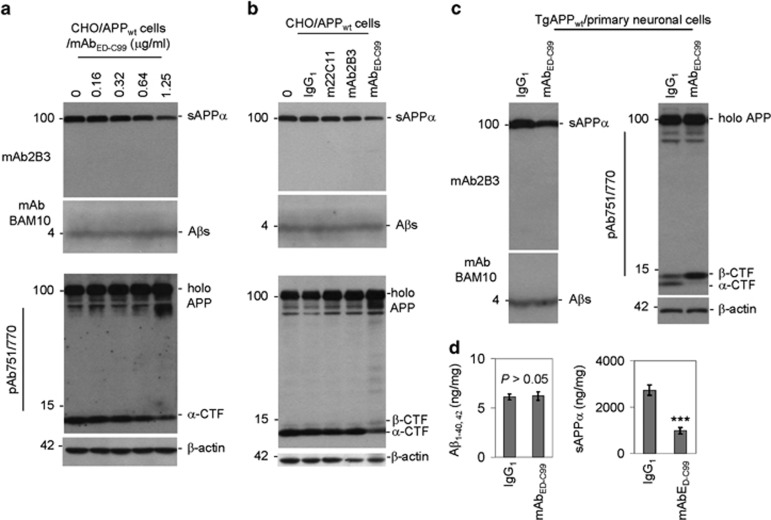
Treatment with mouse monoclonal-specific anti-ED-C99 antibody (mAb_ED-C99_) markedly inhibits *α*-cleavage but promotes *β*-cleavage of human wild-type APP. (**a**) Human wild-type APP stably transfected CHO (CHO/APP_wt_) cells were plated in 24-well plates at 5 × 10^5^/well and treated with mAb_ED-C99_ at 0–1.25 *μ*g/ml as indicated. (**b**) CHO/APP_wt_ cells were treated with mAb_ED-C99_, mAb22C11 (m22C11, recognizes APP_66–81_), or mAb2B3 (specifically and structurally recognizes sAPP*α*, but not full-length APP) antibodies, or isotype IgG_1_ control at 1.25 *μ*g/ml. Immediately after 2 h treatment, cell supernatants were collected for western blotting (WB) analysis of sAPP*α* (using mAb2B3, upper panels) and A*β* secretion (using a monoclonal A*β*_1–12_ antibody BAM10, mAbBAM10, middle panels); cell lysates were prepared for WB analysis of APP processing products (using a polyclonal anti-C-terminal APP_751/770_ antibody, pAb751/770) and *β*-actin (internal control, lower panels). (**c**) Primary neuronal cells were cultured from cortical tissues of 1-day-old TgAPP_wt_ mouse pups and seeded into 24-well-plates at 2 × 10^5^/well for 18 h. These primary neuronal cells were treated with mAb_ED-C99_ or IgG_1_ isotype control at 1.25 *μ*g/ml for 2 h and then cell cultured media were collected for WB analysis of sAPP*α* (left upper panel) and secreted A*β* (left lower panel) levels as indicated; cell lysates were prepared for WB analysis of full-length APP (holo APP), APP-CTFs (right upper panel), and *β*-actin (right lower panel). These WB data are representative of four independent experiments with similar results. (**d**) In addition, the cell culture media were collected from the separated primary cortical neuronal cells following 8 h treatment with mAb_ED-C99_ or IgG_1_ isotype control at 1.25 *μ*g/ml for A*β*_1-40/42_ and sAPP*α*-ELISA. The results were presented as ng of A*β*_40/42_ or sAPP*α* per mg of total intracellular proteins (mean±S.D.; ***P<0.001). These ELISA data are representative of three independent experiments with similar results

Western blotting (WB) analysis demonstrated that, compared with IgG_1_ isotype, 2 h treatment of CHO/APP_wt_ cells with mAb_ED-C99_ dose-dependently inhibits APP *α*-cleavage, as evidenced by the markedly decreased sAPP*α* and *α*-CTF levels (Figure 1a). In contrast, neither mAb22C11 (anti-APP_66–81_ antibody) nor mAb2B3 (sAPP*α*-specific antibody) inhibited the *α*-cleavage of APP (Figure 1b). Most notably, mAb_ED-C99_ shifted APP processing from *α*- to *β*-cleavage, as indicated by decreased sAPP-*α* and *α*-CTF levels in combination with clearly elevated *β*-CTF level (Figure 1b). However, mAb_ED-C99_ did not increase A*β* production, as assessed using mAbBAM10 (recognizes A*β*_1–12_; Figures 1a and b, middle panels).

Consistent with these findings in CHO/APP_wt_ cells, 2 h treatment of primary cultured cortical neurons derived from TgAPP_wt_ mice with mAb_ED-C99_ dramatically inhibited *α*-cleavage but enhanced *β*-cleavage of APP, as indicated by decreased levels of sAPP*α* and *α*-CTF as well as markedly increased *β*-CTF generation, compared with IgG_1_-treated control cells (Figure 1c). In addition, sAPP*α* levels were also significantly decreased with mAb_ED-C99_ treatment (Figure 1d, right panel). However, as seen with CHO/APP_wt_ cells, mAb_ED-C99_ did not significantly alter A*β*_1–40/42_ productions (Figure 1c, left lower panel) and their levels (Figure 1d, left panel). Altogether, these results indicate that specific binding of mAb_ED-C99_ to the ectodomain of *β*-CTF (ED-C99) reduces *α*-secretase processing while increasing *β*-secretase processing of APP.

### F(ab′)_2_ fragment of mAb_ED-C99_ sufficiently inhibits *α*- but promotes *β*-cleavage of human wild-type APP

**Figure 2:**
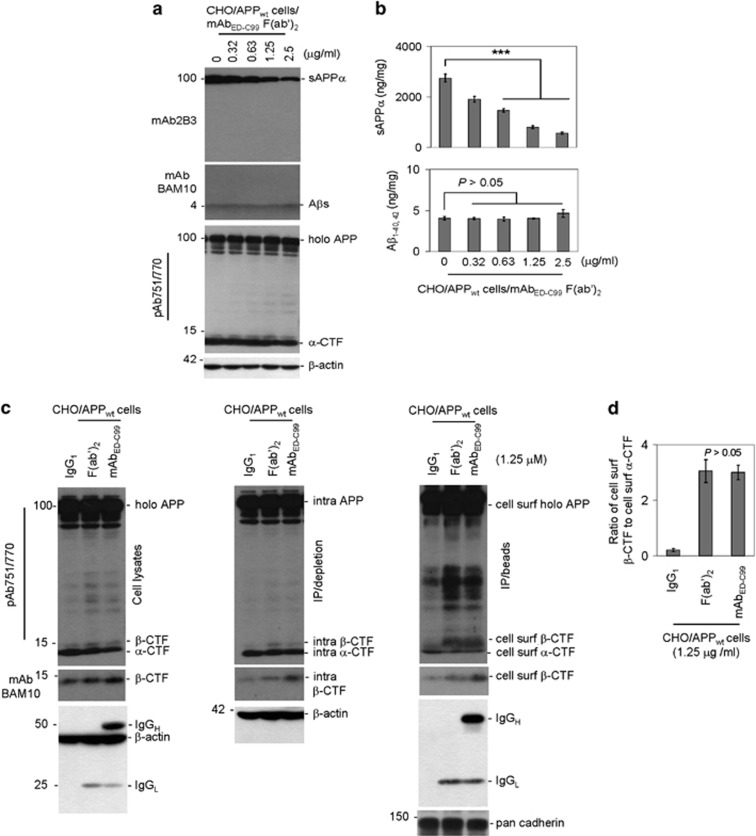
The mAb_ED-C99_ F(ab′)_2_ fragment binding sufficiently enhances human wild-type APP *β*-cleavage. CHO/APP_wt_ cells were treated with F(ab′)_2_ fragment of mAb_ED-C99_ at 0–2.5 *μ*g/ml. (**a**) Immediately following 2 h treatment, cell supernatants were collected for WB analysis of sAPP*α* (using mAb2B3, upper panel), A*β* secretion (using mAbBAM10, middle panel), and APP processing (using pAb751/770, lower panel). (**b**) The secreted sAPP*α* and A*β*_40/42_ levels were analyzed by ELISA and presented as ng of A*β*_1–40/42_ or sAPP*α* per mg of total intracellular proteins (mean±S.D.; ***P<0.001). (**c**) CHO/APP_wt_ cells were treated with mAb_ED-C99_ F(ab′)_2_ fragment, mAb_ED-C99_, or isotype control IgG_1_ at 1.25 *μ*g/ml for 2 h, washed three times with PBS containing CaCl_2_ and MgSO_4_ (PBS-CM), and then cell lysate portions of these cells were directly subjected to WB analysis using pAb751/770 or mAbBAM10 (left panels). The remaining cells were biotinylated with Sulfo-NHS-LC-Biotin dissolved in ice-cold borate buffer, quenched with NH_4_Cl-PBS-CM, and lysed. These cells lysates were then immunoprecipitated using Neutravidin beads. The intracellular (intra) proteins obtained by IP/Neutravidin depletion (middle panels) and the cell surface (cell surf) proteins obtained by IP/Neutravidin precipitation (right panels) were subjected to WB analysis using pAb751/770 and mAbBAM10. (**d**) For WB quantitative analysis, the band density ratio of cell surface *β*-CTF to cell surface *α*-CTF was analyzed and presented as mean±S.D. As indicated, there is no statistically significant difference between mAb_ED-C99_ F(ab′)_2_ fragment and whole mAb_ED-C99_ treatments (*P*>0.05). The data are representative of three independent experiments with similar results

In living cells, the Fc fragment of antibody binds nonspecifically to cell surface Fc receptors. In order to determine whether nonspecific binding of mAb_ED-C99_ to the cell surface Fc receptors is necessary to inhibit APP *α*-processing, we generated the F(ab′)_2_ fragment of mAb_ED-C99_ that lacks the Fc fragment. Consistent with the results obtained with mAb_ED-C99_, 2 h treatment of CHO/APP_wt_ cells with mAb_ED-C99_ F(ab′)_2_ fragment dose-dependently reduced sAPP*α* expression (Figure 2a, top panel) and its levels (Figure 2b, upper panel), while leaving secreted A*β*_1–40/42_ abundances (Figure 2a, middle panel) and their levels (Figure 2b, lower panel) unaltered at the doses examined. Treatment with mAb_ED-C99_ F(ab′)_2_ fragment also slightly reduced secreted *α*-CTF levels (Figure 2a, lower panel), further supporting that specific binding of mAb_ED-C99_ to the extracellular domain of *β*-CTF reduces *α*-cleavage of human wild-type APP. Moreover, mAb_ED-C99_ F(ab′)_2_ fragment also dramatically enhanced cell surface *β*-CTF levels (Figure 2c, right panel) as well as WB band density ratio of cell surface *β*-CTF to cell surface *α*-CTF in CHO/APP_wt_ cells (Figure 2d). The increased level of *β*-CTF is further confirmed using mAbBAM10 that is specific for *β*-CTF but not *α*-CTF (Figure 2c, middle panels).

Most importantly, the monoclonal anti-*β*-actin antibody clearly detected not only *β*-actin but also IgG_1_ heavy and light chains in the whole mAb_ED-C99_-treated condition. In contrast, we only observed IgG_1_ light chain in the mAb_ED-C99_ F(ab′)_2_ fragment-treated condition, confirming the purity of the prepared F(ab′)_2_ fragment. We did not observe IgG_1_ heavy and light chains in the IgG_1_-treated condition, suggesting that both mAb_ED-C99_ F(ab′)_2_ fragment and whole mAb_ED-C99_ can specifically bind to membrane-associated full-length human wild-type APP, whereas control IgG_1_ cannot (Figure 2c, lower panels).

### Specific binding of mAb_ED-C99_ or its F(ab′)_2_ fragment directly inhibits ADAM10-mediated *α*-cleavage of human wild-type APP

**Figure 3:**
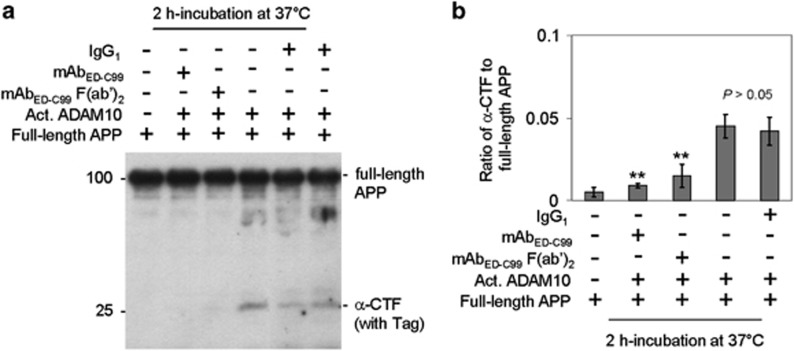
The mAb_ED-C99_ F(ab′)_2_ fragment and mAb_ED-C99_ significantly block ADAM10-mediated *α*-cleavage of recombinant human wild-type APP protein. (**a**) Purified recombinant human wild-type full-length APP protein with Tags (C-terminal MYC/DDK; 260 ng) was incubated with IgG_1_ isotype control, mAb_ED-C99_, or mAb_ED-C99_ F(ab′)_2_ fragment (each at 1 *μ*g) in the presence or absence of activated ADAM10 (Act. ADAM10; 1 unit) in a total volume of 50 *μ*l justified with a buffer. Immediately following 1 h incubation at 37°C, the resulting incubation products were directly subjected to WB analysis using pAb751/770. (**b**) For WB quantitative analysis, band density ratio of *α*-CTF to full-length APP was analyzed and presented as mean±S.D. (**P<0.005). The WB result is representative of three independent experiments with similar results

We hypothesized that the decreased sAPP*α* and *α*-CTF levels elicited by mAb_ED-C99_ or its F(ab′)_2_ fragment might be because of the direct inhibition of ADAM10-mediated *α*-cleavage of human wild-type APP. As expected, compared with IgG_1_-treated control, WB analysis showed that both whole mAb_ED-C99_ and F(ab′)_2_ fragment significantly reduced WB band density ratio of *α*-CTF to full-length human wild-type APP, after incubation of full-length recombinant human wild-type APP protein with active ADAM10 in a cell-free system (Figure 3). These results clearly suggest that the binding of mAb_ED-C99_ or its F(ab')_2_ fragment to ED-C99 blocks ADAM10 from proteolysing human wild-type APP at *α*-cleavage sites.

### Specific binding of mAb_ED-C99_ reduces APP endocytosis while increasing cell surface *β*-CTF

**Figure 4:**
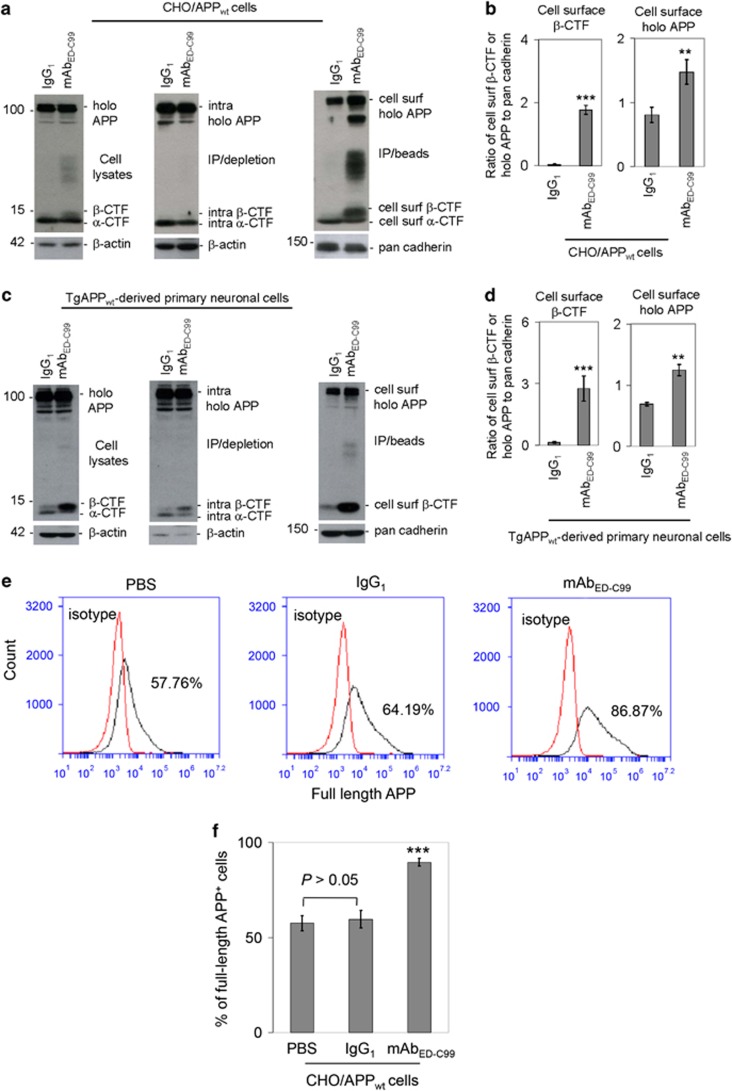
Cell surface *β*-CTF and full-length APP are increased following treatment with mAb_ED-C99_. (**a**) CHO/APP_wt_ cells in 24-well plates (5 × 10^5^/well) were treated with mAb_ED-C99_ or IgG_1_ isotype control at 1.25 *μ*g/ml for 2 h, washed three times with PBS-CM, and then cell lysate portions of these cells were directly subjected to WB analysis using pAb751/770 (left panels). The remaining cells were biotinylated with Sulfo-NHS-LC-Biotin, quenched with NH_4_Cl-PBS-CM, lysed, and immunoprecipitated (IP) using Neutravidin beads. The intracellular proteins obtained by IP/Neutravidin depletion (middle panels) and the cell surface (cell surf) proteins obtained by IP/Neutravidin precipitation (right panels) were subjected to WB analysis using pAb751/770. As shown below each panel, as an internal control, *β*-actin was analyzed for cell lysates and intracellular (intra) proteins and pan cadherin was analyzed for total cell surface proteins. (**b**) For WB quantitative analysis, band density ratios of cell surface *β*-CTF or full-length APP to pan cadherin were analyzed and presented as mean±S.D. (**P<0.01, ***P<0.001). The WB data are representative of three independent experiments with similar results. (**c**) Primary neuronal cells were cultured from cortical tissues of 1-day-old TgAPP_wt_ mouse pups and replated in 24-well plate at 2 × 10^5^/well overnight. These primary neuronal cells were treated with mAb_ED-C99_ or IgG_1_ isotype control at 1.25 *μ*g/ml for 2 h, washed three times with PBS-CM and then cell lysates were directly subjected to WB analysis using pAb751/770 (left panels). The remaining cells were biotinylated, immunoprecipitated with Neutravidin beads, and then subjected to isolation of intracellular and cell surface proteins. The intracellular proteins obtained by IP/Neutravidin depletion (middle panels) and the cell surface (cell surf) proteins obtained by IP/Neutravidin isolation (right panels) were subjected to WB analysis using pAb751/770. (**d**) For WB quantitative analysis, band density ratios of cell surface *β*-CTF or holo APP to pan cadherin were analyzed and presented as mean±S.D. (**P<0.01, ***P<0.001). These WB data are representative of two independent experiments with similar results. (**e**) Flow cytometry analysis of cell surface APP utilizing a rabbit anti-N-terminal APP antibody. (**f**) Percentage of full-length APP-positive cells are presented as mean±S.D. (***P<0.001)

After reaching cell surface via secretory pathway, the matured human wild-type APP molecules could either be predominantly proteolyzed by *α*-secretases or rapidly endocytosed into the early endosomes through endocytosis pathway and then metabolized by *β*- and *γ*-secretases to generate A*β*.^12, 13^ As mAB_ED-C99_ did not alter levels of A*β*, we hypothesized that specific binding to ED-C99 may reduce APP endocytosis. In order to confirm whether mAb_ED-C99_ binding to APP_672–699_ region could modulate human wild-type APP endocytosis, we further assessed the impacts of mAb_ED-C99_ on full-length APP accumulations on the cell surface using the cell surface biotinylation technique.^14^ As compared with IgG_1_ isotype control, WB analysis of biotinylated proteins revealed that 2 h treatment of CHO/APP_wt_ cells with mAb_ED-C99_ markedly increased cell surface full-length APP level (Figure 4a, right panel) as well as significantly enhanced WB band density ratio of cell surface full-length APP to pan cadherin (Figure 4b, right panel). In parallel with these findings in CHO/APP_wt_ cells, 2 h treatment of primary cultured cortical cells derived from TgAPP_wt_ mice with mAb_ED-C99_ also significantly increased cell surface full-length APP level (Figure 4c, right panel) as well as significantly increased WB band density ratio of cell surface full-length APP to pan cadherin (Figure 4d, right panel). The accumulation of cell surface APP was confirmed by flow cytometry that revealed that 2 h treatment of CHO/APP_wt_ cells with mAb_ED-C99_ led to a significant higher percentage of APP-positive cells when compared with IgG_1_ isotype control (Figures 4e and f). These results suggest that specific antibody binding to ED-C99 indeed reduces APP endocytosis. Interestingly, mAb_ED-C99_ also dramatically enhanced cell surface *β*-CTF levels (Figures 4a and c, right panels) as well as significantly increased WB band density ratio of *β*-CTF to pan cadherin (Figures 4b and d, left panels) in both CHO/APP_wt_ cells and primary cultured cortical cells derived from TgAPP_wt_ mice. The markedly increased cell surface *β*-CTF level in both cell types following treatment with mAb_ED-C99_ were further confirmed by mAbBAM10, an antibody specifically recognizing *β*-CTF but not *α*-CTF (Supplementary Figure S2). Taken together, these findings suggest that the specific bindings of mAb_ED-C99_ to APP_672–699_ region inhibits human wild-type APP *α*-cleavage, while also blocking human wild-type APP endocytosis and promoting its *β*-cleavage and/or cell surface accumulation of *β*-CTF.

### Specific mAb_ED-C99_ binding enhances the colocalization of human wild-type APP with cholesterol

**Figure 5:**
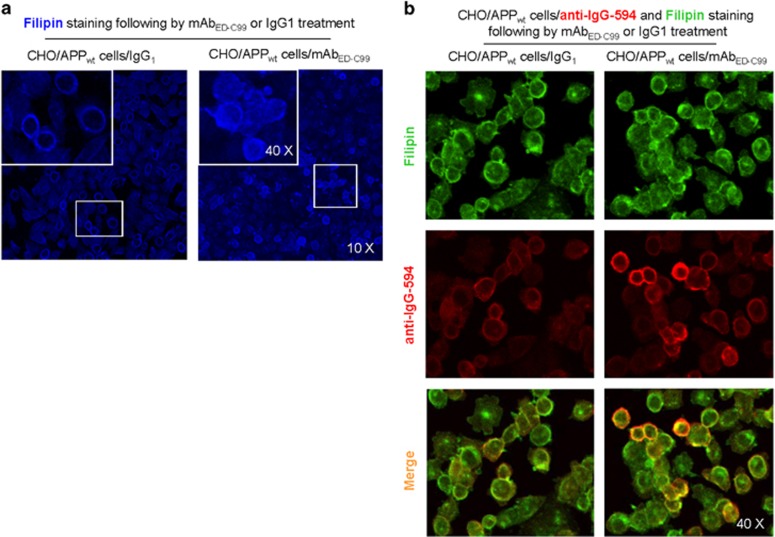
Colocalization of human wild-type APP with cholesterol in CHO/APP_wt_ cells after mAb_ED-C99_ treatment. (**a**) CHO/APP_wt_ cells were plated to 8-well slide chamber and then, after overnight incubation, the cells were treated with mAb_ED-C99_ or IgG_1_ isotype control for 2 h. These cells were stained by Filipin in strict accordance with the manufacturer's instructions of the cholesterol assay kit. (**b**) After Filipin staining and washing, some of these cells were permeabilized with 0.05% Triton X-100 for 5 min, washed, and stained with rabbit anti-APP-C-terminal antibody overnight at 4°C. Alexa Fluor 594 Donkey anti-rabbit IgG was used to detect APP signals. Confocal images were taken by Olympus fluoview FV1000 laser scanning confocal microscope (Tokyo, Japan)

As growing evidence have suggested that cholesterol is of particular importance in regulating APP processing,^15, 16, 17^ favoring *β*-cleavage and amyloidogenic processing, we further determined colocalization of human wild-type APP with cholesterol in CHO/APP_wt_ cells following 2 h mAb_ED-C99_ treatment. Although 2 h treatment with mAb_ED-C99_ increased the cholesterol level on both cellular and subcellular plasma membranes (as indicated by dispersed fillipin staining, Figure 5a), this enhanced human wild-type APP with cholesterol colocalization was primarily observed on cell surface but rarely in intracellular compartments (Supplementary Figure S3), indicating that the mAb_ED-C99_-induced human wild-type APP with cholesterol colocalization is cell surface specific. In addition, merged image of cholesterol staining with fillipin (green) and rabbit anti-APP-C-terminal antibody labeling with anti-IgG-594 (red) revealed a higher colocalization of human wild-type APP with cholesterol in mAb_ED-C99_-treated CHO/APP_wt_ cells compared with IgG_1_ isotype-treated control cells (Figure 5b). This cell surface colocalization of APP with cholesterol may further reduce APP *α*-cleavage and favor *β*-cleavage and cell surface *β*-CTF accumulation.

### LRP1-CT overexpression reverses mAb_ED-C99_-mediated cell surface accumulation of *β*-CTF

**Figure 6:**
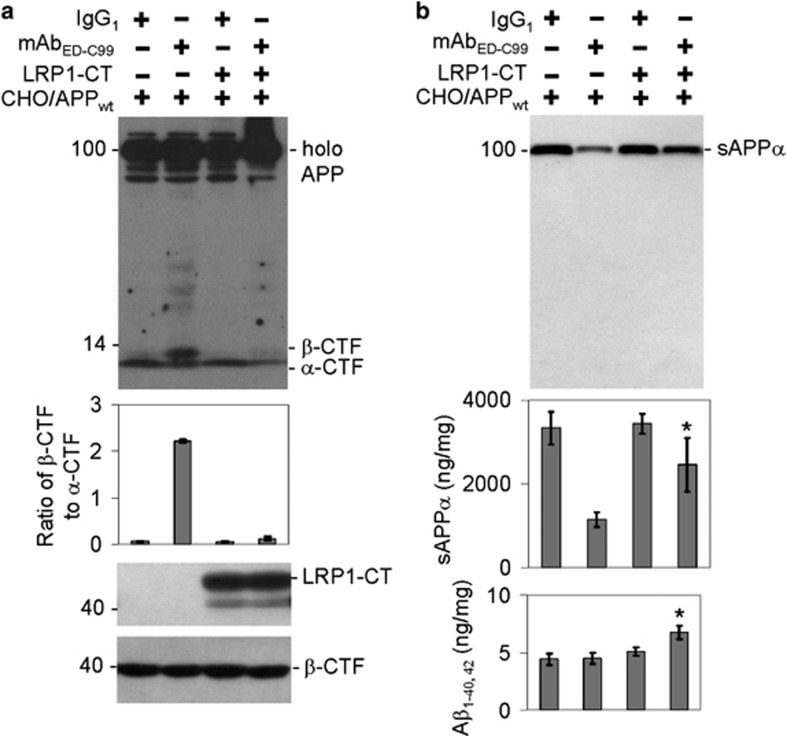
Overexpressing LRP1-CT markedly restores human wild-type APP endocytosis in CHO/APP_wt_/LRP1-CT cells. CHO/APP_wt_/LRP1-CT or CHO/APP_wt_ cells were treated with mAb_ED-C99_ for 2 h. (**a**) Full-length APP and *α*/*β*-CTFs were examined by WB analysis. (**b**) The secreted sAPP*α* and A*β*_1–40/42_ levels in conditioned media were measured by WB analysis and ELISA. The band ratio of *β*-CTF to *α*-CTF and sAPP*α* ELISA results (ng of sAPP*α* per mg of total proteins) are presented as mean±S.D. These data are representative of three independent experiments with similar results (*P<0.05)

APP endocytosis is known to be mediated by binding to the low-density lipoprotein receptor-related protein-1 (LRP1). To further confirm that mAb_ED-C99_ reduces APP endocytosis while enhancing cell membrane *β*-CTF accumulation, CHO/APP_wt_ cells overexpressing the cytoplasmic tail of LRP1 (LRP1-CT) were exposed with mAb_ED-C99_ for 2 h. Both WB and enzyme-linked immunosorbent assay (ELISA) analyses clearly suggested that although 2 h treatment of CHO/APP_wt_ cells with mAb_ED-C99_ promoted cell surface *β*-CTF accumulation as compared with IgG_1_ treatment (Figure 6a, upper panel, lanes 1 and 2), this change was dramatically reversed by overexpressed LRP1-CT (Figure 6a, upper panel, lanes 3 and 4). Surprisingly, LRP1-CT overexpression also significantly but partially reversed the mAb_ED-C99_-mediated decrease of the secreted sAPP*α* abundances in the conditioned media (Figure 6b, upper panel, lanes 3 and 4) as well as its levels (Figure 6b, middle panel). Most interestingly, the secreted A*β*_1–40/42_ levels in the conditioned media were significantly elevated in CHO/APP_wt_/LRP1-CT cells following the reversed inhibition of APP endocytosis (Figure 6b, lower panel).

### mAb_ED-C99_ promotes *β*-cleavage of human wild-type APP *in vivo*

**Figure 7:**
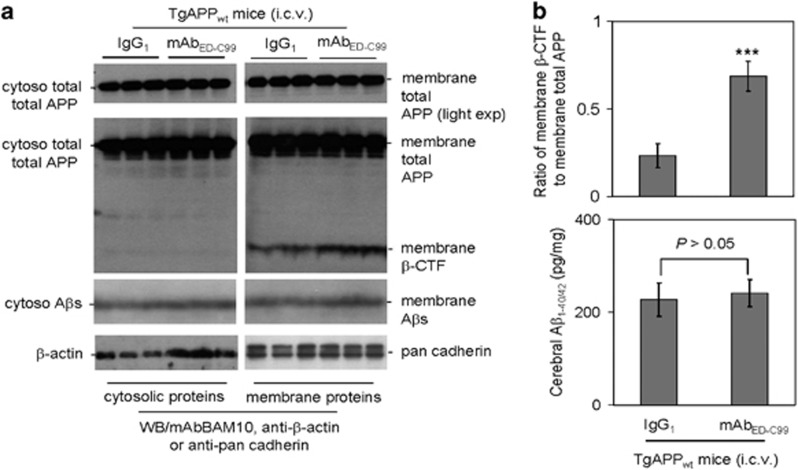
The mAb_ED-C99_ promotes APP *β*-secretase processing *in vivo*. TgAPP_wt_ female mice at 8 months of age were treated with mAb_ED-C99_ or control IgG_1_ at 5 *μ*g/mouse by intracerebroventricular (i.c.v.) injection and killed 24 h after the treatment (*n*=6). (**a**) Cytosolic- (left panels) and membrane-associated proteins (right panels) prepared from mouse brain homogenates were subjected to WB analysis for APP processing. (**b**) For WB quantitative analysis, band density ratio of membrane-associated *β*-CTF to membrane-associated total APP was analyzed (upper panel). A*β*_40/42_ was also analyzed by ELISA (lower panel, *n*=6). The results are presented as pg of A*β*_40/42_ per mg of total intercellular proteins (mean±S.D.; ***P<0.001)

Eight-month-old TgAPP_wt_ female mice were treated with mAb_ED-C99_ or isotype IgG_1_ as negative control via intracerebroventricular (i.c.v.) injection. At 24 h after treatment, we found that WB band ratio of membrane-bound *β*-CTF to total APP in the mAb_ED-C99_-treated group was significantly higher than that in the IgG_1_-treated control group (Figure 7a, middle panels, and Figure 7b, upper panel). Most interestingly, consistent with our *in vitro* data, mAb_ED-C99_ did not alter the levels of A*β* species (Figure 7a, lower panels). A*β* ELISA analysis of brain homogenates also confirmed that A*β*_1–40/42_ species were not significantly changed in the mAb_ED-C99_-treated group compared with IgG_1_ control group (Figure 7b, lower panel). In addition, 8-month-old 5 × FAD female transgenic mice (Tg6799 line) were treated with mAb_ED-C99_ or isotype IgG_1_ as negative control via i.c.v. injection. At 24 h after treatment, we found that immunohistochemical staining also disclosed comparable amount of *β*-amyloid plaques in retrosplenial cortex (RSC), entorhinal cortex (EC), and hippocampus (H) regions of 5 × FAD transgenic mouse brains (Supplementary Figure S4).

## Discussion

Mutations in *APP* gene cause early onset of autosomal-dominant AD.^3, 18, 19, 20^ Relative to their wild-type homologs, the English (APP_677_ H→R) and Tottori (APP_678_ D→N) substitutions accelerate the kinetics of A*β* secondary structure change from statistical coil→*α*/*β*→*β* and produce oligomer size distributions skewed to higher order that are more toxic to cultured neuronal cells than wild-type oligomers.^21^ The Icelandic APP_673_ mutation (A→V) affects APP processing, resulting in enhanced A*β* production (quantity) and formation of amyloid fibrils *in vitro* (quality).^8^ In contrast, alternative APP_673_ mutation (A→T) results in an ∼40% reduction in the formation of amyloidogenic peptides and therefore protects against AD and cognitive decline in the elderly.^22^ Thus, these pathogenic mutations located in the APP_672_ to APP_699_ region of APP ectodomain (also named ED-C99) encompassing APP *α*- and *β*-cleavage sites can alter *β*-cleavage and A*β*-related AD pathology. However, unlike autosomal-dominant fAD, sAD patients generally lack mutations of *APP* gene. Therefore, the mechanisms underlying pathogenesis of sAD are still far from clarification. Here, we hypothesized that modifications (either physical or functional interactions) of regions close to APP *α*- and *β*-cleavage sites by either endogenous or exogenous molecules may yield similar impacts on human wild-type APP processing to what is observed with APP mutations.

Cell surface *α*-cleavage is the predominant processing pathway for human wild-type APP. In the present study, we found that the specific binding of the APP ED-C99 region with mAb_ED-C99_ dose-dependently blocks *α*- but promotes *β*-cleavage of human wild-type APP (Figure 1). These effects were further confirmed by another specific antibody, 4G8, recognizing the ED-C99 domain (with epitope at APP_688–695_, data not shown). In contrast, N-terminal APP antibody 22C11 (epitope of APP_66–81_) or sAPP*α*-specific antibody mAb2B3 (epitope of APP_672–688_, absence of affinity to full-length APP) showed no effect on human wild-type APP processing. This lack of impact of mAb22C11 and mAb2B3 further indicates that the effect of mAb_ED-C99_ on human wild-type APP processing is region specific. In addition, these modulations on human wild-type APP *α*/*β*-cleavage induced by mAb_ED-C99_ can be recapitulated by using the F(ab′)_2_ fragment of mAb_ED-C99_ (Figure 2). As F(ab′)_2_ lacks the nonspecific Fc binding subunit, these results confirm that mAb_ED-C99_-induced modification of APP processing is ED-C99 region specific. Our present study further reveals that this *α*-cleavage inhibiting activity is due, in part, to the direct physical blocking of ADAM10 (Figure 3).

Previous studies have suggested that intracellular trafficking is pivotal for human wild-type APP proteolysis.^12, 13^ In contrast, non-amyloidogenic processing occurs mainly at the cell surface, where *α*-secretases are present. Amyloidogenic processing involves transit through the endocytic organelles, where APP encounters *β*- and *γ*-secretases.^13, 15^ In our present study, mAb_ED-C99_ enhanced cell surface APP accumulation, which was confirmed by both WB and flow cytometry analyses, indicating that ED-C99 binding reduces the APP endocytic pathway. Blockage of human wild-type APP *α*-cleavage by mAb_ED-C99_ may consequently lead to an elevated *β*-cleavage that has been confirmed by dramatically increased *β*-CTF. This suggests that ED-C99 may bind and trap APP in the membrane (or endosomes in the process of fusing with the plasma membrane), making the proteolytic sites for ADAM10 inaccessible, and thus shifting the proteolysis of APP in the plasma membrane or endosomes to *β*-secretase cleavage.

This proposed blockage of human wild-type APP *α*-cleavage by mAb_ED-C99_ may consequently lead to an elevated *β*-cleavage that has been confirmed by the dramatically increased *β*-CTF levels on the cell surface (Figure 4). Our findings suggest that this elevated *β*-cleavage can be because of the cell surface APP with cholesterol colocalization (Figure 5). Indeed, growing evidence has demonstrated that cholesterol is of particular importance in regulating *α*- and *β*-cleavage of APP.^15, 16, 17^ Cholesterol can bind to APP_696–699_, an N-loop structure at the end of the ED-C99 region, thereby inhibiting *α*-cleavage while favoring *β*-cleavage of APP. In contrast to *β*-secretase, previous studies have indicated that *γ*-secretase cleavage of C99 appears to occur primarily in rafts located in the endosomes.^12, 23, 24, 25, 26, 27, 28, 29^ Thus, the specific binding of mAb_ED-C99_ to APP inhibits APP *α*-cleavage and endocytosis, thereby locking APP on the cell surface to be cleaved by *β*-secretase while functionally blocking *γ*-secretase from producing A*β*. This would explain how the mAb_ED-C99_ promoted cell surface *β*-CTF accumulation with a concordant rise in A*β* protein levels.

In addition to cholesterol, it has also been shown that LRP1 plays a crucial role in APP processing.^30, 31, 32^ Multiple APP processing steps may be modulated by LRP1, most of which can be attributed to its ability to bind and endocytose APP via its LRP1-CT domain. In the absence of LRP1, APP internalization rates are reduced by ∼50%, while cell surface APP and APP-CTFs accumulate.^32^ In order to verify the possibility that mAb_ED-C99_ alters APP processing by reducing endocytosis, CHO/APP_wt_/LRP1-CT cells were treated with mAb_ED-C99_. Our results demonstrated a market restoration of the inhibited endocytosis, as shown by the decreased cell surface *β*-CTF level (Figure 6a) and increased A*β* levels (Figure 6b). Surprisingly, the reduction of sAPP*α* by mAb_ED-C99_ was attenuated by LRP1-CT overexpression (Figure 6b), suggesting that LRP1 may block the mAb_ED-C99_ binding on APP_672–699_ region and subsequently release APP from mAb_ED-C99_-mediated inhibition of *α*-secretase.

To confirm whether the promotion of cell surface *β*-CTF accumulation induced by binding of mAb_ED-C99_ to ED-C99 *in vitro* could be repeated *in vivo*, TgAPP_wt_ mice at 8 months of age were treated (i.c.v.) with mAb_ED-C99_ or IgG_1_ as negative control. We found that mAb_ED-C99_ treatment significantly increased cell surface *β*-CTF, as indicated by elevated ratio of *β*-CTF/total APP (Figure 7b). Similar to our *in vitro* study, mAb_ED-C99_ treatment also yielded no significant changes of A*β* production compared with the IgG_1_ control. Although the amyloid cascade hypothesis is potentially viable in cases of genetic mutation-caused autosomal-dominant fAD, accumulating evidence suggests that it may not apply in the vast majority of patients with late-onset sAD lacking mutations of APP/presenilin genes.

These works suggest several possibilities in terms of defining a possible etiology and treatment target for both fAD and sAD. First, A*β*-related plaques is one relatively common finding in the non-demented elderly. In fact, recent studies have demonstrated that accumulation of *β*-CTF may have direct deleterious effects on cognitive function. For example, inhibition of *β*-site amyloid cleaving enzyme (BACE) rescued synaptic/memory deficits in a mouse model of familial Danish dementia.^33^ However, *γ*-secretase inhibition worsened memory deficits in these mice that correlated with increased levels of APP *α*/*β*-CTFs in synaptic fractions of the hippocampus.^34^ In another report, prolonged (8 days) treatment with *γ*-secretase inhibitors (GSIs) produced no positive effects on memory deficits of older Swedish APP transgenic mice, but induced cognitive deficits in young Swedish APP transgenic mice or wild-type mice.^35^ Indeed, a recent phase III clinical trial with the GSI Semagacestat was halted because it worsened clinical measures of cognition and the ability to perform activities of daily living. These results suggest that *β*-CTF rather than A*β* may be more directly responsible for causing cognitive impairment associated with AD and that GSIs may worsen cognitive impairment by enhancing the accumulation of *β*-CTF. Our results also point to a need to identify, epidemiologically, the presence or absence of mAb_ED-C99_-like antibodies or proteins that may increase with age and correlate with onset of AD-like signs and symptoms. In addition, this work suggests that such an antibody or protein could reduce *α*-secretase function and thus generation of sAPP*α*, a peptide that is neuroprotective.^36, 37, 38^

In summary, the present study verifies our hypothesis that ED-C99 region (APP_672–699_) is critical for human wild-type APP processing. Specific binding of this region will direct inhibit APP *α*-secretase activity and reduce APP endocytosis, thereby enhancing cell surface *β*-CTF accumulation. These effects may have deleterious effects of cognition, and this should be further explored in future studies. Modifications (physical of functional interactions) of this region with either exogenous or endogenous molecules will affect human wild-type APP processing, potentially enhancing or ameliorating the development of AD (Supplementary Figure S5).

## Materials and Methods

### Antibodies

Sterile and low-endotoxin antibodies were used, including anti-C-terminal human sAPP*α*-specific antibody 2B3 (mAb2B3; IBL, Minneapolis, MN, USA), APP_66–81_ antibody 22C11 (m22C11; Roche, Basel, Switzerland), specific monoclonal ED-C99 antibody (mAb_ED-C99_, 6E10; Covance, Emeryville, CA, USA), APP C-terminal antibody pAb751/770 (EMD Biosciences, La Jolla, CA, USA), specific monoclonal antibody BAM10 (Sigma-Aldrich, St. Louis, MO, USA), *β*-actin antibody (Sigma-Aldrich), and anti-pan cadherin antibody (AbCam, Cambridge, MA, USA). The sAPP*α*-specific 2B3 antibody was further characterized in our i*n vitro* and *in vivo* systems, indicating that this antibody recognizes neither A*β* nor full-length APP. The mAb_ED-C99_ F(ab′)_2_ fragment was generated using F(ab′)_2_ preparation kit (Thermo Fisher Scientific, Waltham, MA, USA) according to the manufacturer's instructions.

### Cell culture and treatment

CHO cells engineered to express wild-type human APP (CHO/APP_wt_) were kindly provided by Dr. Steanie Hahn and Dr. Sascha Weggen (University of Heinrich Heine, Düsseldorf, Germany). Plasmid PLHCX-LRP1-CT was a generous gift from Dr. David Kang (University of South Florida, Tampa, FL, USA). LRP1-CT was subcloned to PCDNA vector by PCR and restrict enzyme *Hin*dIII and *Not*I digestion. The primers were used as follows: forward 5′-AGCTCGTTTAGTGAACCGTCAGATC-3′ and reverse 5′-ATGCGGCCGCTCATGCCAAGGGGTCCCCTATC-3′. Stable cell lines were generated by transfection of PCDNA-LRP1-CT to CHO/APP_wt_ cells and single colony was picked up after G418 administration. These cells were maintained in Dulbecco's modified Eagle's medium (DMEM) with 10% fetal bovine serum, 1 mM sodium pyruvate, and 100 U/ml penicillin/streptomycin.^39^ For TgAPP_wt_ mouse-derived cortical neurons, cerebral cortices isolated from 1-day-old TgAPP_wt_ mice were mechanically dissociated in trypsin (0.25%) individually after incubation for 15 min at 37°C. Cells were collected after centrifugation at 1200 × *g*, suspended in DMEM supplemented with 10% fetal calf serum, 10% horse serum, uridine (33.6 *μ*g/ml, Sigma-Aldrich), and fluorodeoxyuridine (13.6 *μ*g/ml, Sigma-Aldrich) and seeded in 24-well collagen-coated culture plates at 2.5 × 10^5^ cells per well. After reaching confluence (∼70–80%), cells were treated with mAb_ED-C99_ at 0–1.25 *μ*g/ml for 2 h. In additional experiments, cells were treated with mAb22C11 or mAb2B3 or mAb_ED-C99_ F(ab′)_2_ fragment at 1.25 *μ*g/ml.

### Transgenic APP_wt_ mice and i.c.v. injection

Transgenic wild-type B6.Cg-Tg (PDGFB-APP) 5Lms/J strain (TgAPP_wt_) female mice and 5 × FAD transgenic female mice (Tg6799 line) were purchased from the Jackson Laboratory (Bar Harbor, ME, USA). All mice were housed and maintained in the Animal Facility of College of Medicine at University of South Florida. At 8 months of age, both mice were anesthetized using isoflurane (chamber induction at 4–5% isoflurane, intubation and maintenance at 1–2%). After reflexes were checked to ensure that mice were unconscious, they were positioned on a stereotaxic frame (Stoelting Lab Standard, Wood Dale, IL, USA) with ear-bars positioned and jaws fixed to a biting plate. The ED-C99-specific antibody mAb_ED-C99_ and isotype control IgG_1_ were dissolved in sterile distilled water at a concentration of 1 *μ*g/*μ*l. mAb_ED-C99_ and control IgG_1_ (5 *μ*l) were injected into the left lateral ventricle with a microsyringe at a rate of 1 *μ*l/min with the coordinates (bregma: −0.6 mm anterior/posterior, +1.2 mm medial/lateral, and −3.0 mm dorsal/ventral) according to our previous methods.^36, 40^ The needle was left in place for 5 min after injection before being withdrawn. At 24 h after i.c.v. injections, animals were killed with isofluorane and brain tissues were collected. All dissected brain tissues were rapidly frozen for analysis. All experiments involving mice were performed in compliance with the US Department of Health and Human Services Guide for the Care and Use of Laboratory Animals and in accordance with the guidelines of the University of South Florida Institutional Animal Care and Use Committee.

### Cell surface biotinylation

Confluent cell cultures in dishes were washed three times with phosphate-buffered saline (PBS)-CM. Cells were biotinylated with 0.5 mg/ml Sulfo-NHS-LC Biotin dissolved in ice-cold borate buffer for 30 min at 4°C. Biotin was changed twice during the 30-min incubation period. Biotinylation was quenched with three 50 mM NH_4_Cl-PBS-CM washes followed by two PBS washes. Cells were harvested and lysed in NP-40 buffer and protein concentration was determined via BCA. Equal amounts of protein were immunoprecipitated using Neutravidinagarose beads overnight at 4°C. Proteins were eluted from the Neutravidinagarose beads by heating at 95°C for 10 min.

### WB analysis

Briefly, for WB analysis, cultured cells were lysed in ice-cold lysis buffer. Proteins were separated using 10% gel, transferred to 0.2-*μ*m nitrocellulose membranes (Bio-Rad, Hercules, CA, USA) and visualized using standard immunoblotting protocol. All antibodies were diluted in Tris-buffered saline (TBS) containing 5% (w/v) nonfat dry milk. Blots were developed using the Luminol reagent (Thermo Fisher Scientific) and densitometric analysis was performed as described previously, using a Fluor-S MultiImager with Quantity One software (Bio-Rad).^40, 41^

### Flow cytometry analysis

CHO/APP_wt_ cells were plated in six-well plate and treated overnight with mAb_ED-C99_ or IgG_1_ isotype control. The cells were then detached by trypsin, stained with mAb_ED-C99_ and Alexa Fluor 488-conjugated donkey anti-mouse IgG (Invitrogen, Gaithersburg, MD, USA) for 30 min on ice, washed with Flow buffer, and analyzed by Accuri C6 Flow Cytometer (Rochester, MN, USA).

### Filipin staining

After treatment, the cells were fixed and Filipin staining was performed following the manufacturer's instructions of the cholesterol assay kit (AbCam).

### ELISA

Total A*β* species, including A*β*_40/42_, in cell conditioned media and brain homogenates were detected by A*β*_1–40/42_ ELISA kits (Invitrogen, Carlsbad, CA, USA) according to the manufacturer's instructions. The sAPP*α* level in cell conditioned media was determined using sAPP*α* ELISA kit (IBL). A*β* and sAPP*α* levels are represented as ng/mg of total cellular protein.

### Statistical analysis

Data are expressed as mean±S.D. of *n* independent experiments. Comparison between two groups was performed by Student's *t*-test. *P*<0.05 was considered statistically significant.

## Abbreviations

A*β*: amyloid-*β*
APP: amyloid precursor protein
AD: Alzheimer's disease
ADAM10: a disintegrin and a metalloproteinase 10
BACE: *β*-site amyloid cleaving enzyme
CHO/APP_wt_ cells: Chinese hamster ovary cells stably transfected with human wild-type APP
CTF: C-terminal fragments
DMEM: Dulbecco's modified Eagle's medium
EC: entorhinal cortex
ED-*β*-CTF: ectodomain of *β*-C-terminal fragment
ELISA: enzyme-linked immunosorbent assay
fAD: familial AD
GSI: *γ*-secretase inhibitor
H: hippocampus
TgAPP_wt_: human wild-type APP transgenic
i.c.v.: intracerebroventricular
LRP1: low density lipoprotein receptor-related protein-1
LRP1-CT: cytoplasmic tail of LRP1
PBS: phosphate-buffered saline
RSC: retrosplenial cortex
sAD: sporadic AD
sAPP*α*: soluble APP*α*
TBS: Tris-buffered saline
WB: western blotting
