# Supplementary material for: Specific antibody binding to the APP672–699 region shifts APP processing from α- to β-cleavage
Source: Cell Death Dis. 2014 Aug 14;5(8):e1374–. doi: 10.1038/cddis.2014.336 (PMC4454311; doi:10.1038/cddis.2014.336)
Supplement: Supplementary Information [file cddis2014336x1.pdf]

Supplementary Information

Specific antibody binding to the $APP_{672\text{-}699}$ region shifts APP processing from α- to β-cleavage

Song Li[1,6,8], Juan Deng[1,7,8], Huayan Hou[1,8], Jun Tian[1], Brian Giunta[2,3], Yanjiang Wang[7], Darrell Sawmiller[1,2], Adam Smith[4], Paul R. Sanberg[4], Demian Obregon[1,2], Takashi Mori[5] and Jun Tan [*,1,2]

[1]Rashid Laboratory for Developmental Neurobiology, Silver Child Development Center, Department of Psychiatry and Behavioral Neurosciences, Morsani College of Medicine, University of South Florida, Tampa, Florida, USA; [2]James A. Haley Veterans' Hospital, Tampa, Florida, USA; [3]Neuroimmunology Laboratory, Department of Psychiatry and Behavioral Neurosciences, Morsani College of Medicine, University of South Florida, Tampa, Florida, USA; [4]Center of Excellence for Aging and Brain Repair, Department of Neurosurgery and Brain Repair, Morsani College of Medicine, University of South Florida, Tampa, Florida, USA; [5]Departments of Biomedical Sciences and Pathology, Saitama Medical Center and Saitama Medical University, Kawagoe, Saitama, Japan; [6]Center for Translational Research of Neurology Diseases, First Affiliated Hospital, Dalian Medical University; [7]Department of Neurology, Daping Hospital, the Third Military Medical University, Chongqing, China

Running title: Binding on $APP_{672\text{-}699}$ promotes APP β-cleavage

[8]These authors contributed equally to this work.

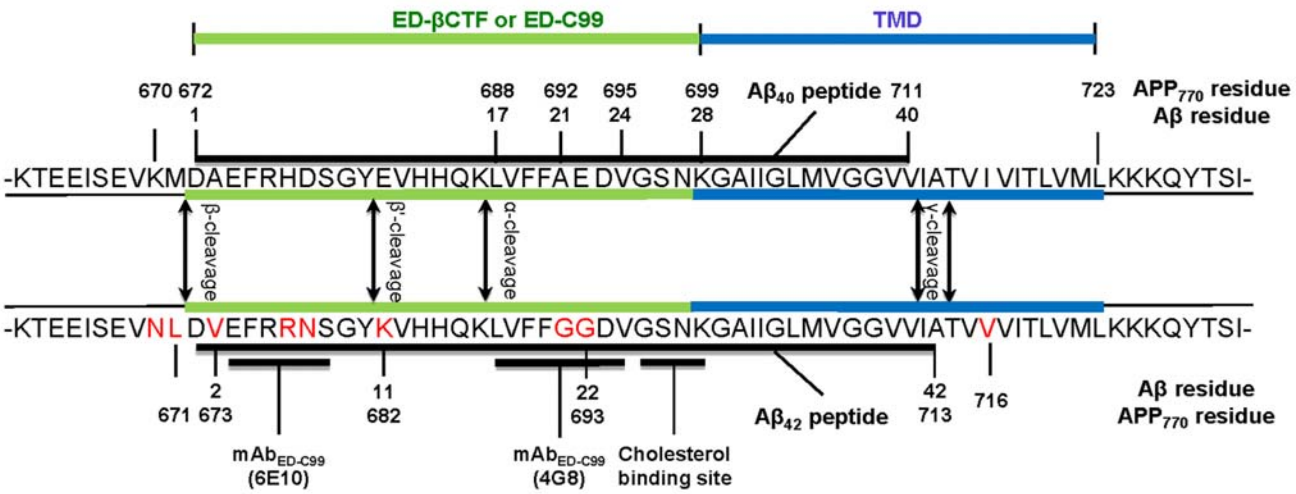

Supplemental Figure S1 A schematic diagram of the β-amyloid precursor protein (APP) proteolytic cleavage sites and mutations. The sites of α-, β- and γ-secretase–mediated cleavage are indicated with arrows. The trans-membrane domain (TMD) of APP is highlighted in blue, and ectodomain (ECD) of APP in green. γ-cleavage produces a pool of Aβ fragments that vary in length and hydrophobicity. The mutations (indicated in red) around α- and β-cleavage sites either increase the total Aβ production (670/671, 682, 692), alter Aβ biophysical properties (677, 678, 693), or affect the Aβ spectrum in both quantitative and qualitative ways (673, 716).

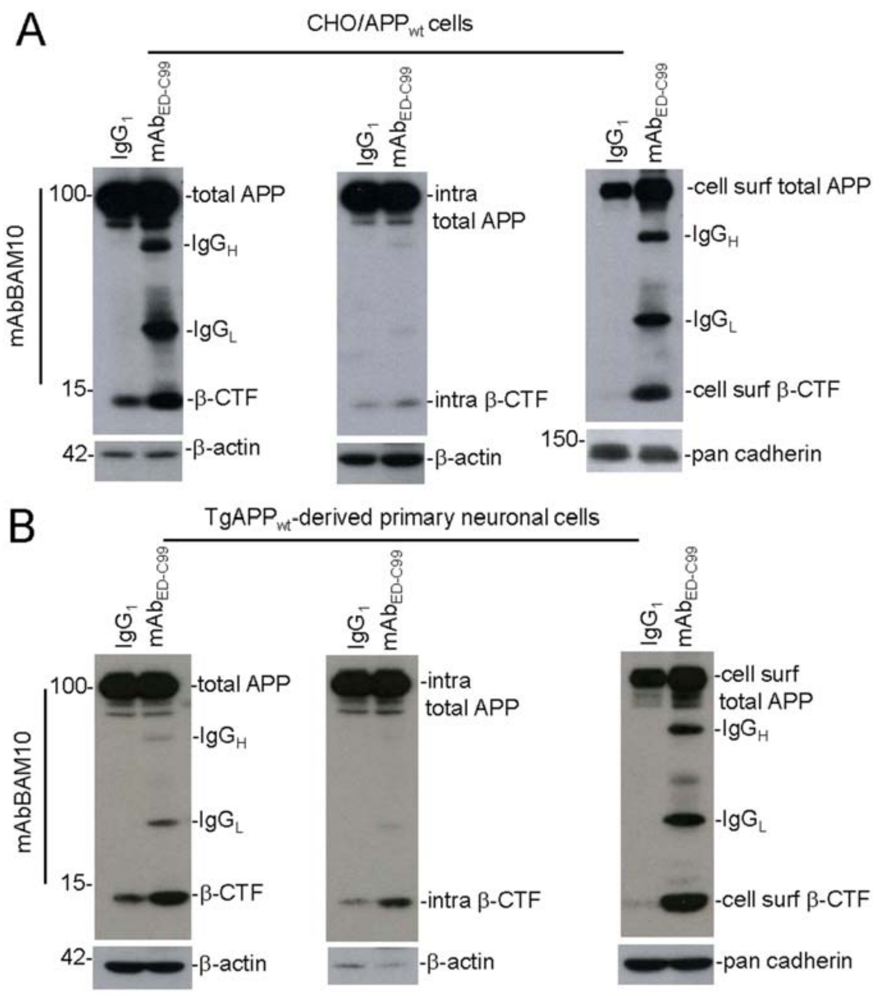

Supplemental Figure S2 Increased cell surface β-CTF is further confirmed by a β-CTF specific antibody. (**A**) CHO/APP_{wt} cells in 24-well plates (5×10^5/well) were treated with $mAb_{ED\text{-}C99}$ or IgG_1 isotype control at 1.25 μg/ml for 2 h, washed three times with PBS containing $CaCl_2$ and $MgSO_4$ (PBS-CM), and then cell lysate portions of these cells were directly subjected to WB analysis using a β-CTF specific antibody, mAbBAM10. The remaining cells were biotinylated with Sulfo-NHS-LC-Biotin dissolved in ice-cold borate buffer and quenched with NH_4Cl-PBS-CM and lysed. These cell lysates were then immunoprecipitated (IP) using Neutravidin beads. The intracellular proteins obtained by IP/Neutravidin depletion (middle panels) and the cell surface (cell surf) proteins obtained by IP/Neutravidin precipitation (right panels) were subjected to WB analysis using mAbBAM10. (**B**) $TgAPP_{wt}$-derived primary

neuronal cells were cultured from cortical tissues of one-day-old $TgAPP_{wt}$ mouse pups and re-plated in 24-well plate at 2×10^5/well overnight. These primary cultured neuronal cells were treated with mAb_{ED-C99} or IgG_1 isotype control at 1.25 μg/ml for 2 h, washed three times with PBS-CM, and then cell lysates were directly subjected to WB analysis using mAbBAM10. The remaining cells were biotinylated, immunoprecipitated with Neutravidin beads and subjected to WB analysis using mAbBAM10. These WB data are representative of four independent experiments with similar results.

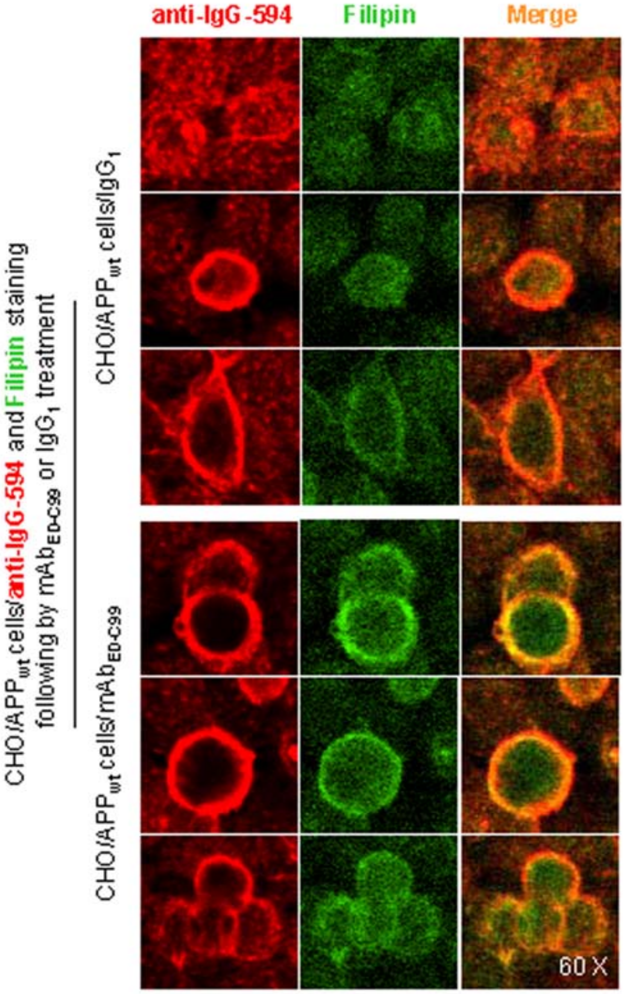

Supplemental Figure S3 Increased co-localization of APP with cholesterol is cell surface specific. CHO/APP_{wt} cells were plated to 8-well slide chamber and then, after overnight

incubation, the cells were treated with mAb_{ED-C99} or IgG_1 control at 1.25 μg/ml for 2 h. Two hours after treatment, these cells were stained by filipin, permeabilized and counterstained with rabbit anti-APP-C-terminal antibody 4°C for overnight, as described for Figure 5. Alexa Fluor® 594 Donkey anti-rabbit IgG was used to detect APP signal and images taken with a confocal microscopy.

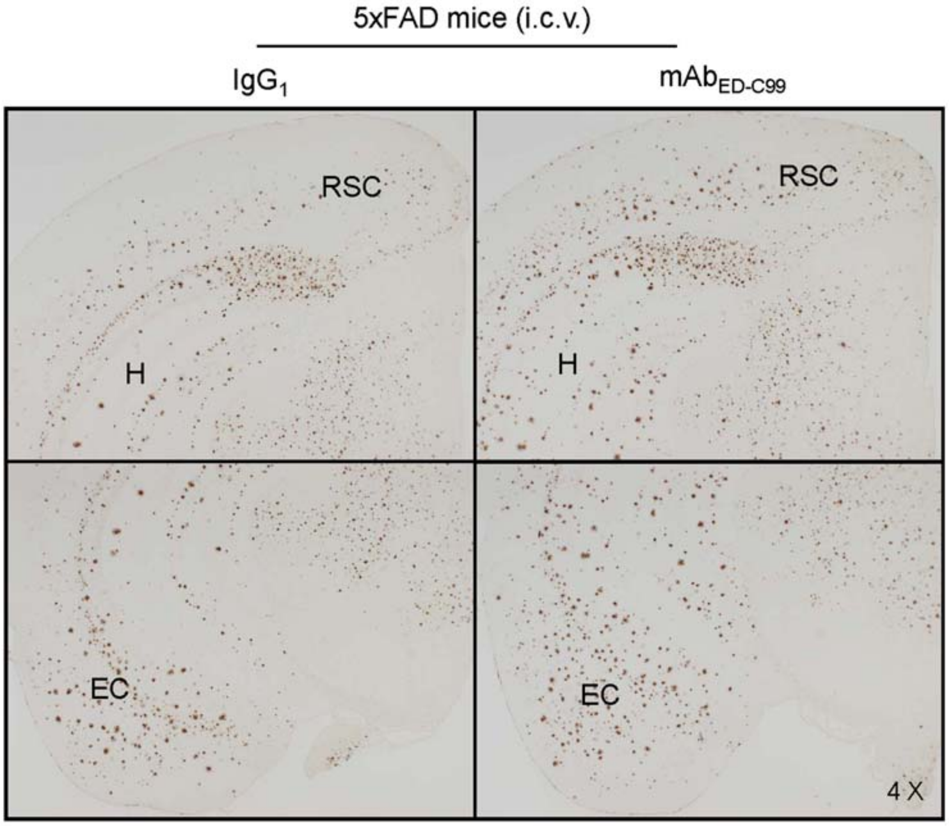

Supplemental Figure S4 Intracerebroventricular (i.c.v.) injection of mAb_{ED-C99} yields no increase Aβ deposits. Eight-month-old 5×FAD female mice (n = 3) were treated with mAb_{ED-C99} or isotype IgG_1 as negative control *via* i.c.v. injection daily for 5 days (5 μg/mouse). The immunohistochemical staining using an anti-$A\beta_{17-26}$ antibody (4G8) indicated no changes of β-

amyloid plaques in retrosplenial cortex (RSC), entorhinal cortex (EC), and hippocampus (H) regions of 5×FAD mouse brain.

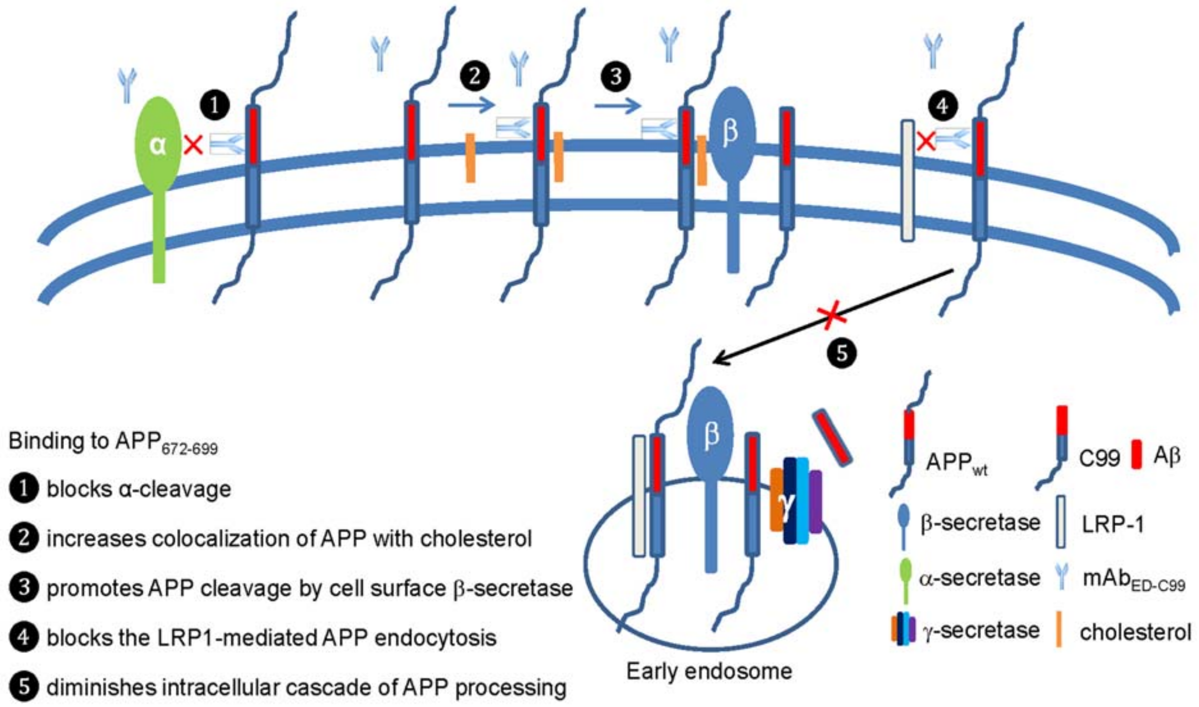

Supplemental Figure S5. Illustration of effects of mAb$_{\text{ED-C99}}$ binding to APP$_{672\text{-}699}$ on wild-type APP processing. APP is primarily processed by α-secretase on the cell membrane surface leading to sAPPα release, or processed while co-localized with cholesterol on the cell membrane surface by β-secretase, leading to C99 production. In addition, APP can be endocytosed for β-secretase cleavage and endocytosed C99 is further processed by γ-secretase to yield Aβ. As shown, mAb$_{\text{ED-C99}}$ can (1) directly block α-secretase activity, (2) increase co-localization of APP with cholesterol, (3) promote APP to be cleaved by cell surface β-secretase, (4) block LRP1-mediated APP endocytosis, and thereby, (5) diminish intracellular cascade of APP processing.
